# Serum Magnesium Levels Across Clinical Stages of Alcoholic Liver Disease: A Cross-Sectional Study From South India

**DOI:** 10.7759/cureus.110388

**Published:** 2026-06-07

**Authors:** Ligin Lavichan, Ramesh Bala Arivazhagan, Selvamuthukumaran S, Prabakaran Vaithinathan, Sridurga Mattyvanan

**Affiliations:** 1 Department of General Medicine, Vinayaka Mission’s Medical College and Hospital, Vinayaka Mission’s Research Foundation (Deemed to be University), Karaikal, IND; 2 Department of Biochemistry, Vinayaka Mission’s Medical College and Hospital, Vinayaka Mission’s Research Foundation (Deemed to be University), Karaikal, IND

**Keywords:** alcoholic liver disease, biomarkers, cross-sectional studies, liver cirrhosis, magnesium, prognosis

## Abstract

Background and objective: Alcoholic liver disease (ALD) involves progressive hepatic dysfunction and frequent electrolyte disturbances, but the relationship between serum magnesium and clinically staged ALD remains unclear. This study aimed to explore the relationship between the two with established markers of disease severity.

Materials and methods: In this hospital‑based cross‑sectional study, 80 adults with confirmed ALD were enrolled at a tertiary center in Karaikal, TN, IND, between January and December 2025. Patients were classified as non‑cirrhotic ALD, compensated cirrhosis, or decompensated cirrhosis. Clinical evaluation, laboratory tests (including serum magnesium), viral serology, ultrasonography, and the model for end-stage liver disease (MELD) and Maddrey’s discriminant function (DF) scores were obtained. Prognostic assessment was performed using the MELD, Maddrey’s DF, and the age-bilirubin-INR-creatinine (ABIC) scores.

Results: Most patients had non‑cirrhotic ALD (53.8%) or decompensated cirrhosis (40.0%); nearly all were male. With advancing stages, hemoglobin, platelets, sodium, albumin, and coagulation indices worsened, and MELD and DF scores increased significantly. Serum magnesium showed a progressive, borderline non‑significant decline (p=0.071).

Conclusion: Conventional biochemical markers and prognostic scores robustly reflected ALD severity, while serum magnesium trended downward across stages, suggesting a potential adjunctive role rather than a standalone severity marker.

## Introduction

Alcoholic liver disease (ALD) remains one of the leading causes of preventable morbidity and mortality worldwide, reflecting the pervasive impact of harmful alcohol use on population health and healthcare systems [1]. The liver is central to ethanol metabolism, and chronic excessive alcohol intake drives a spectrum of hepatic injury influenced by nutritional status, host genetics, coexisting metabolic or inflammatory conditions, and cumulative alcohol exposure. Alcoholic liver disease encompasses a continuum from simple steatosis and alcoholic hepatitis to progressive fibrosis, cirrhosis, and decompensated end‑stage liver disease, frequently accompanied by multi‑organ dysfunction and high short‑term mortality [2].

The global burden of ALD is rising, with alcohol accounting for a substantial proportion of liver‑related hospitalizations and deaths in both high‑income and low‑ and middle‑income countries [3]. In resource‑constrained settings such as India, delayed presentation, limited access to early hepatology care, persistent social stigma, and high rates of malnutrition among individuals with alcohol use disorder further shift the clinical spectrum toward advanced disease at first contact [4].

Pathologically, ALD progresses through overlapping stages rather than a fixed linear sequence. Simple steatosis, often clinically silent and reversible with abstinence, is characterized by triglyceride accumulation in hepatocytes driven by altered lipid metabolism and redox imbalance [5]. With continued alcohol exposure, hepatocellular ballooning, neutrophil‑predominant inflammation, and varying necrosis define alcoholic hepatitis, which in severe forms carries a high risk of short‑term mortality. Chronic inflammation and fibrogenesis ultimately culminate in cirrhosis, portal hypertension, and hepatic decompensation manifested by ascites, variceal bleeding, encephalopathy, and hepatorenal syndrome [6].

Clinical assessment of ALD integrates history, physical examination, biochemical tests, imaging, and prognostic scores. Conventional laboratory abnormalities include elevated aminotransferases (typically with an aspartate aminotransferase (AST) > alanine transaminase (ALT) profile), hyperbilirubinemia, hypoalbuminemia, and prolonged prothrombin time/INR, reflecting hepatocellular injury and impaired synthetic function [7]. Ultrasonography, a widely available modality in low‑resource settings, helps detect steatosis, textural changes, nodularity, splenomegaly, ascites, and features of portal hypertension, though its sensitivity in early disease is limited. Composite prognostic indices such as the model for end-stage liver disease (MELD), Maddrey’s discriminant function (DF), and the age-bilirubin-INR-creatinine (ABIC) score are routinely used to stratify short-term mortality risk and guide decisions regarding intensive therapy or transplant referral [8-10].

Chronic alcohol consumption causes profound systemic metabolic derangement beyond the liver, altering carbohydrate, lipid, and protein metabolism and profoundly disturbing micronutrient homeostasis. Individuals with AUD often have poor dietary intake, impaired intestinal absorption, increased urinary losses, and altered hepatic handling of key vitamins and minerals [11]. Among these, magnesium is particularly important because it serves as a cofactor in more than 300 enzymatic reactions, including those involved in ATP generation, nucleic acid synthesis, and oxidative phosphorylation. Magnesium also stabilizes cell membranes, modulates ion channels, and regulates inflammatory and immune responses. In the liver, adequate intracellular magnesium supports mitochondrial integrity, limits oxidative stress, and maintains hepatocyte resilience, processes that are critically perturbed in alcohol‑induced injury [12].

Hypomagnesemia has been associated with elevated pro‑inflammatory cytokines such as interleukin‑6 and tumor necrosis factor‑α, higher oxidative stress markers, and more severe forms of alcoholic hepatitis and cirrhosis. In patients with AUD and early ALD, lower magnesium levels have been linked to heavier drinking patterns, greater inflammatory activity, and markers of hepatocyte apoptosis and necrosis [11,12].

The present hospital‑based cross‑sectional study was conducted in a South Indian tertiary care center to examine serum magnesium levels across well‑defined clinical stages of ALD and to explore their relationship with established markers of disease severity. Patients were systematically classified into non‑cirrhotic ALD, compensated cirrhosis, and decompensated cirrhosis based on clinical and ultrasonographic criteria, and disease burden was quantified using conventional biochemical parameters, MELD, Maddrey’s DF, and ABIC scores [8-10].

## Materials and methods

Study design and setting

This is a hospital‑based cross‑sectional observational study that was conducted in the Department of General Medicine at Vinayaka Mission's Medical College and Hospital (VMMCH), Karaikal, TN, IND. The study period extended from January 2025 to December 2025. Approval was obtained from the Institutional Ethics Committee of Vinayaka Mission's Medical College (approval no. IEC/VMMCH/2021/APR/14). Participation was entirely voluntary, and refusal to participate did not affect the standard of care received. Written informed consent was obtained from all participants, and confidentiality of patient information was maintained throughout the study.

The study population comprised adult patients with a diagnosis of ALD attending the outpatient and inpatient services of the Department of General Medicine during the study period. Both newly diagnosed and previously known cases of ALD were eligible for inclusion. Patients were included if they met all of the following criteria: age greater than 18 years; history of alcohol consumption for more than five years; average daily alcohol intake ≥ 40 g on at least four days per week; clinically and/or investigatively established diagnosis of ALD, with or without complications.

Exclusion criteria were liver disease attributable to non‑alcoholic causes (e.g., viral hepatitis, autoimmune liver disease, or non‑alcoholic fatty liver disease); congenital liver disorders or primary hepatocellular pathology unrelated to alcohol use; coexisting conditions independently associated with magnesium depletion (such as malabsorption syndromes or relevant endocrine/metabolic disorders); severe renal impairment; history of dyselectrolytemia in the absence of alcohol consumption; current or recent (within the preceding three months) use of magnesium supplementation; and known hepatocellular carcinoma.

Sample size and sampling technique

Sample size was calculated using the formula 4pq/d2, with p taken as 20% (anticipated proportion of patients with hypomagnesemia), q = 1 − p, absolute precision (d) of 5%, and a 95% confidence level, yielding a minimum sample size of 72. After inflating for an anticipated 10% non‑response or exclusion rate, the final target sample size was set at 80, and this target was achieved. A consecutive sampling strategy was employed, whereby all eligible patients presenting during the study period were screened and enrolled until the required sample size was reached. The sample size calculation formula used in the study is typically applicable for estimating prevalence in community-based studies.

Data collection

Potentially eligible patients were identified in outpatient clinics and inpatient wards. The study objectives, procedures, potential risks, and benefits were explained in the local language, and written informed consent was obtained from each participant or from a legally acceptable representative when necessary. Venous blood samples were obtained for laboratory investigations, and abdominal ultrasonography was performed according to clinical indication and study protocol.

Clinical staging of ALD

Patients were categorized into three mutually exclusive clinical stages based on the composite of clinical and ultrasonographic findings. (1) Non‑cirrhotic ALD was confirmed by ultrasonography that lacked features of cirrhosis, with findings confined to fatty liver or early inflammatory changes and absence of ascites or portal hypertension. (2) Compensated cirrhosis was seen with ultrasonographic evidence of cirrhosis features of coarse hepatic echotexture, nodular liver surface, and/or splenomegaly; and no clinical or imaging features of decompensation (no ascites, portal hypertension, hepatic encephalopathy, or gastrointestinal/variceal bleeding). (3) Decompensated cirrhosis is when ultrasonographic morphology is consistent with cirrhosis, and there is a presence of at least one of the following decompensating events: ascites, portal hypertension, hepatic encephalopathy, or variceal/gastrointestinal bleeding. Alcoholic hepatitis was not treated as a separate anatomical stage; instead, its severity was quantified using established prognostic scores (Maddrey’s DF and MELD [9,10]) within the above staging framework.

Laboratory and Imaging Investigations

All participants underwent the following investigations as part of routine clinical care and study assessment. Complete blood count (hemoglobin, total leukocyte count, platelet count, and erythrocyte sedimentation rate); liver function tests (total, direct, and indirect bilirubin); AST; ALT; alkaline phosphatase (ALP); total protein; and serum albumin. Renal function tests included serum urea and creatinine, coagulation profile (prothrombin time (PT) and international normalized ratio (INR)), serum electrolytes (sodium, potassium, and chloride), and serum magnesium concentration measured using the laboratory’s standard automated methodology. Viral serology was undertaken for hepatitis B surface antigen (HBsAg) and anti‑hepatitis C virus (HCV) antibody. Ultrasonography of the abdomen and pelvis was conducted to document liver size and texture, splenic size, presence of ascites, and sonographic features of portal hypertension. Serum magnesium levels were subsequently categorized into normal and graded hypomagnesemia (mild, moderate, and severe) according to predefined cut‑offs used in the study.

Severity scoring systems

Disease severity within each clinical stage was objectively quantified using validated prognostic indices, namely the MELD score, Maddrey’s DF, and ABIC score [8-10]. Patients were additionally stratified into standard risk categories for MELD, DF (severe ≥32 vs. non-severe), and ABIC (low, intermediate, and high risk). These scores were used to examine the relationship between graded disease severity and serum magnesium levels. Severe alcoholic hepatitis was defined by Maddrey’s DF score ≥32. A MELD score >20 and ABIC score categories were interpreted using previously validated standard cut-off values described in earlier studies.

Statistical analysis

Data were analyzed using SPSS Statistics version 27 (IBM Corp., Armonk, NY, USA). Continuous variables were summarized as mean ± standard deviation or median (interquartile range), depending on the distribution assessed by the Shapiro-Wilk test. Categorical variables were expressed as frequencies and percentages. Comparisons of continuous variables across the three clinical stages of ALD were performed using ANOVA with appropriate post hoc tests for normally distributed data or the Kruskal‑Wallis test for non‑normally distributed data. Associations between categorical variables were evaluated using the chi‑square test or Fisher’s exact test, as appropriate. 

## Results

The study cohort comprised 80 patients, more than half of whom had non‑cirrhotic ALD, while approximately two-fifths had decompensated cirrhosis, and only a small minority had compensated cirrhosis. Age showed a clear gradient in severity: non‑cirrhotic disease predominated in younger age groups, whereas decompensated cirrhosis was most frequent among patients aged 50 years and above and universal in those aged 70 years or older (Table 1).

**Table 1:** Demographic and clinical stage characteristics of patients with ALD (total n = 80) ALD: Alcoholic liver disease

Variable		Non‑cirrhotic ALD (n = 43)	Compensated cirrhosis (n = 5)	Decompensated cirrhosis (n = 32)	Total (n = 80)
Age group, n (%)	20-29 years	4 (9.3)	1 (20.0)	0 (0.0)	5 (6.3)
	30-39 years	12 (27.9)	1 (20.0)	3 (9.4)	16 (20.0)
	40-49 years	14 (32.6)	2 (40.0)	8 (25.0)	24 (30.0)
	50-59 years	9 (20.9)	1 (20.0)	13 (40.6)	23 (28.8)
	60-69 years	4 (9.3)	0 (0.0)	10 (31.3)	14 (17.5)
	≥70 years	0 (0.0)	0 (0.0)	6 (18.8)	6 (7.5)
Sex, n (%)	Male	42 (97.7)	5 (100.0)	32 (100.0)	79 (98.8)
	Female	1 (2.3)	0 (0.0)	0 (0.0)	1 (1.2)

Progressive worsening of hematological and coagulation parameters was evident with advancing clinical stage. Hemoglobin and platelet counts were significantly lower in decompensated cirrhosis compared with non‑cirrhotic disease, reflecting anemia of chronic disease, occult blood loss, and hypersplenism in advanced portal hypertension. Prothrombin time and INR were markedly prolonged in decompensated cirrhosis, consistent with impaired hepatic synthetic function and increased bleeding risk in these patients. Renal involvement appeared as rising blood urea levels with increasing severity, whereas creatinine and random blood sugar values varied without statistically significant differences across stages, suggesting early or subclinical renal compromise in many patients (Table 2).

**Table 2:** Hematological, coagulation, glycemic, and renal parameters by clinical stage of ALD (total n = 80) A p-value < 0.05 was considered statistically significant, and a p-value < 0.001 was considered highly statistically significant. Data are presented as mean ± SD for continuous variables. ALD: Alcoholic liver disease; INR: International normalized ratio

Parameter	Non‑cirrhotic ALD (n = 43) Mean ± SD	Compensated cirrhosis (n = 5) Mean ± SD	Decompensated cirrhosis (n = 32) Mean ± SD	H statistic (Kruskal–Wallis)	p value
Hemoglobin (g/dL)	11.8 ± 2.9	11.5 ± 2.1	8.7 ± 2.4	20.78	< 0.001
Total leukocyte count (×10³/mm³)	9.8 ± 5.1	7.2 ± 2.8	10.9 ± 7.6	4.64	0.098
Platelet count (×10³/mm³)	228 ± 112	198 ± 98	148 ± 89	12.69	0.002
Prothrombin time (s)	14.8 ± 3.2	15.6 ± 3.9	26.4 ± 18.7	9.53	0.009
INR	1.3 ± 0.3	1.4 ± 0.3	2.6 ± 1.6	9.61	0.008
Random blood sugar (mg/dL)	128 ± 78	142 ± 65	138 ± 92	1.95	0.377
Urea (mg/dL)	24.1 ± 12.6	28.4 ± 14.2	38.7 ± 22.3	8.03	0.018
Creatinine (mg/dL)	0.8 ± 0.3	0.9 ± 0.4	1.2 ± 0.9	2.32	0.314

Markers of liver excretory and synthetic function deteriorated significantly with the advancing stage of ALD. Total bilirubin concentrations rose sharply in decompensated cirrhosis, indicating substantial cholestasis and hepatocellular dysfunction in this group. Serum albumin levels showed a stepwise decline from non‑cirrhotic disease to decompensated cirrhosis, reflecting progressive loss of hepatic protein synthetic capacity and contributing to edema and ascites. The AST activity increased significantly across stages, whereas ALT did not differ markedly, consistent with the characteristic AST‑predominant enzyme pattern of alcoholic liver injury (Table 3).

**Table 3:** Liver function tests, enzymes, and serology by clinical stage of ALD (total n = 80) Data are presented as mean ± SD for continuous variables and frequency (n) with percentage (%) for categorical variables. A p-value < 0.05 was considered statistically significant. ALD: Alcoholic liver disease; AST: Aspartate aminotransferase; ALT: Alanine aminotransferase; HBsAg: Hepatitis B surface antigen; HCV: Hepatitis C virus

Parameter	Non‑cirrhotic ALD (n = 43) Mean ± SD or n (%)	Compensated cirrhosis (n = 5) Mean ± SD or n (%)	Decompensated cirrhosis (n = 32) Mean ± SD or n (%)	H statistic (Kruskal–Wallis) / test	p value
Total bilirubin (mg/dL)	2.31 ± 1.89	2.85 ± 1.42	8.92 ± 7.12	43.41	< 0.001
AST (U/L)	78 ± 68	92 ± 74	128 ± 89	9.15	0.010
ALT (U/L)	48 ± 42	56 ± 51	68 ± 52	4.36	0.113
Albumin (g/dL)	3.68 ± 0.62	3.45 ± 0.71	2.78 ± 0.68	27.43	< 0.001
HBsAg positive, n (%)	0 (0.0)	0 (0.0)	0 (0.0)	NA	NA
Anti‑HCV positive, n (%)	0 (0.0)	0 (0.0)	0 (0.0)	NA	NA

Electrolyte disturbances intensified with increasing severity of ALD, most notably as significant declines in serum sodium and chloride among patients with decompensated cirrhosis, compatible with dilutional hyponatremia and advanced portal hypertension. Mean serum magnesium values showed a gradual numerical reduction from non-cirrhotic to decompensated stages; however, differences narrowly missed statistical significance, likely reflecting limited power in the small compensated group. Although the overall distribution of magnesium severity categories and the presence of hypomagnesemia did not reach significance, mild hypomagnesemia was more frequent in compensated and decompensated cirrhosis than in non‑cirrhotic disease, suggesting a trend toward worsening magnesium status with progression (Table 4).

**Table 4:** Electrolytes, serum magnesium, ultrasonographic features, and magnesium related categories by clinical stage of ALD (total n = 80) Data are presented as mean ± SD for continuous variables and frequency (n) with percentage (%) for categorical variables. A p-value < 0.05 was considered statistically significant. ALD: Alcoholic liver disease

Variable	Non‑cirrhotic ALD (n = 43)	Compensated cirrhosis (n = 5)	Decompensated cirrhosis (n = 32)	Test statistic / exact test	p-value
Sodium (mEq/L), mean ± SD	136.2 ± 4.1	135.8 ± 5.2	132.4 ± 6.8	H = 9.78	0.008
Potassium (mEq/L), mean ± SD	3.9 ± 0.6	3.8 ± 0.7	3.7 ± 0.8	H = 3.57	0.168
Chloride (mEq/L), mean ± SD	100.1 ± 4.3	98.5 ± 5.1	97.2 ± 6.4	H = 18.14	< 0.001
Magnesium (mg/dL), mean ± SD	2.12 ± 1.12	1.78 ± 0.52	1.69 ± 0.78	H = 5.28	0.071
Coarse echotexture present, n (%)	0 (0.0)	5 (100.0)	32 (100.0)	Fisher’s exact	< 0.001
Surface nodularity present, n (%)	0 (0.0)	5 (100.0)	32 (100.0)	Fisher’s exact	< 0.001
Ascites present, n (%)	0 (0.0)	0 (0.0)	30 (93.8)	Fisher’s exact	< 0.001
Portal hypertension present, n (%)	0 (0.0)	0 (0.0)	28 (87.5)	Fisher’s exact	< 0.001
Magnesium severity category, n (%)				Fisher’s exact	0.220
Normal	32 (74.4)	3 (60.0)	18 (56.3)	NA	NA
Mild hypomagnesemia	9 (20.9)	2 (40.0)	13 (40.6)	NA	NA
Moderate hypomagnesemia	1 (2.3)	0 (0.0)	1 (3.1)	NA	NA
Severe hypomagnesemia	1 (2.3)	0 (0.0)	0 (0.0)	NA	NA
Hypomagnesemia present, n (%)	11 (25.6)	2 (40.0)	14 (43.8)	Fisher’s exact	0.191

All three prognostic systems demonstrated strong and consistent associations with clinical stages. Mean MELD scores more than doubled from non‑cirrhotic disease to decompensated cirrhosis, with half of the decompensated patients falling into the highest MELD category (≥20), indicating substantial short‑term mortality risk. The DF scores showed a similar pattern, with severe DF (≥32) present in three-quarters of decompensated cases but in fewer than one-fifth of non-cirrhotic and compensated patients, highlighting the heavy burden of severe alcoholic hepatitis in advanced cirrhosis. The ABIC scores rose progressively across stages, and high‑risk ABIC categories were concentrated in decompensated cirrhosis, while low‑risk scores predominated in non‑cirrhotic ALD, reinforcing the discriminatory value of these validated indices for stratifying prognosis in ALD (Table 5).

**Table 5:** Prognostic scores (MELD, Maddrey’s DF, ABIC) and risk categories by clinical stage of ALD (total n = 80) Data are presented as mean ± SD for continuous variables and frequency (n) with percentage (%) for categorical variables. A p-value < 0.05 was considered statistically significant, and a p-value < 0.001 was considered highly statistically significant. ALD: Alcoholic liver disease; MELD: Model for end‑stage liver disease; DF: Discriminant function; ABIC: Age-bilirubin-INR-creatinine

Variable	Non-cirrhotic ALD (n = 43)	Compensated cirrhosis (n = 5)	Decompensated cirrhosis (n = 32)	Test statistic/exact test	p-value
MELD score, mean ± SD	8.9 ± 4.8	9.1 ± 3.9	20.7 ± 8.1	H = 38.20	< 0.001
MELD category, n (%)				Fisher’s exact	< 0.001
<10	28 (65.1)	3 (60.0)	2 (6.3)	NA	NA
10-14	11 (25.6)	1 (20.0)	6 (18.8)	NA	NA
15-19	3 (7.0)	1 (20.0)	8 (25.0)	NA	NA
≥20	1 (2.3)	0 (0.0)	16 (50.0)	NA	NA
DF score, mean ± SD	19.4 ± 22.1	22.6 ± 18.4	124.8 ± 170.5	H = 28.60	< 0.001
DF category, n (%)				Fisher’s exact	< 0.001
Non-severe (<32)	39 (90.7)	4 (80.0)	8 (25.0)	NA	NA
Severe (≥32)	4 (9.3)	1 (20.0)	24 (75.0)	NA	NA
ABIC score, mean ± SD	6.5 ± 1.8	7.9 ± 2.4	11.2 ± 4.1	H = 38.42	< 0.001
ABIC risk category, n (%)				Fisher’s exact	< 0.001
Low risk (<6.71)	24 (55.8)	2 (40.0)	3 (9.4)	NA	NA
Intermediate (6.71-8.99)	15 (34.9)	1 (20.0)	8 (25.0)	NA	NA
High risk (≥9)	4 (9.3)	2 (40.0)	21 (65.6)	NA	NA

## Discussion

The present study evaluated 80 patients with ALD across clearly defined clinical stages and demonstrated a distribution with a predominance of non-cirrhotic ALD and decompensated cirrhosis and relatively few patients identified at the compensated stage. Most patients belonged to the non-cirrhotic group, while decompensated cirrhosis formed the next largest category and compensated cirrhosis accounted for only a small fraction, reflecting the tendency for individuals to present either early with biochemical or imaging abnormalities or late with complications.

Age‑stratified analysis revealed a strong and biologically plausible association between advancing age and increasing disease severity. Younger participants predominantly had non‑cirrhotic ALD, whereas decompensated cirrhosis became progressively more common from the fifth decade onward and was universal beyond 70 years of age, reflecting the cumulative effect of prolonged alcohol exposure and duration‑dependent risk of cirrhosis and its complications. Mogalapu et al. [13] similarly found that older patients clustered in more severe Child‑Pugh classes, and Winrich et al. [14] reported that longer duration of heavy drinking and higher cumulative intake were closely linked to advanced disease and poorer outcomes.

A male predominance was observed, with all patients being male and all cirrhosis cases occurring in men. This aligns with prior Indian and international cohorts in which men represent 80% to 85% or more of hospitalized alcohol‑related liver disease cases, as reported by Mogalapu et al. [13] and Pavuluri et al. [15]. Although women develop ALD at lower alcohol doses and drinking histories, social and cultural factors likely contribute to the under‑representation of female patients in clinical cohorts.

Hematological alterations showed a pattern of progressive derangement with advancing stage. Hemoglobin levels fell significantly from non‑cirrhotic disease to decompensated cirrhosis, and platelet counts were substantially lower in decompensated patients, while total leukocyte counts did not differ significantly between groups. Similar trends have been documented in prior work, including the study by Mogalapu et al. [13], where hemoglobin and platelet counts declined across increasing Child‑Pugh classes and MELD strata. Coagulation parameters further highlighted the severity gradient: both prothrombin time and INR were markedly prolonged in decompensated cirrhosis, reflecting severe dysfunction and impaired hepatic production of clotting factors [16].

Random blood sugar values did not differ significantly across stages of ALD, which may reflect the heterogeneity of metabolic alterations in cirrhosis [17]. Serum urea showed a significant increase with disease severity. Serum creatinine did not show much variation in the study. This indicates early involvement of the renal system in severe liver disease [17].

Electrolyte analysis confirmed the pattern of dilutional hyponatremia and associated chloride reduction in decompensated cirrhosis, reflecting non‑osmotic vasopressin release, impaired free water clearance, and advanced portal hypertension. Potassium levels did not show a consistent trend, likely because of competing influences of diuretic therapy, dietary intake, vomiting, and renal adaptation [18]. Mean serum magnesium showed a gradual decline from non‑cirrhotic disease to decompensated cirrhosis, and hypomagnesemia was more frequent in advanced stages, although differences did not reach statistical significance, probably due to limited sample size and exclusion of other strong magnesium‑wasting conditions. Rani et al. [19] reported significantly lower magnesium levels in more severe cirrhosis and negative correlations with Child‑Pugh or MELD scores.

Total bilirubin increased markedly with disease severity, reflecting worsening excretory function and cholestasis. The AST levels were significantly higher in decompensated cirrhosis, while ALT remained relatively stable, reaffirming the AST‑predominant transaminase profile characteristic of alcohol‑related injury. Serum albumin declined significantly across stages, indicating progressive loss of synthetic capacity and contributing to edema, ascites, and poorer prognostic stratification, in line with classical Child‑Pugh criteria and multiple ALD cohorts. All patients were negative for HBsAg and anti‑HCV, confirming alcohol as the primary etiologic factor in this cohort and eliminating viral co‑infection as a confounder of both liver disease severity and magnesium status [20].

Ultrasonography correlated closely with clinical staging. Coarse hepatic echotexture and surface nodularity were confined to cirrhotic patients, while ascites and ultrasound markers of portal hypertension predominantly occurred in the decompensated group. These findings align with established imaging criteria for cirrhosis and portal hypertension and corroborate the accuracy of the staging framework employed. Importantly, ultrasound was able to differentiate non‑cirrhotic from cirrhotic disease and to distinguish compensated from decompensated states when combined with clinical features, demonstrating its utility as a non‑invasive tool in resource‑limited settings [21]. As expected, ultrasonographic features such as coarse echotexture, surface nodularity, ascites, and portal hypertension differed significantly across groups, as these parameters formed part of the predefined criteria used for clinical staging of ALD. Therefore, these findings should be interpreted primarily as confirmation of appropriate group classification rather than independent discriminatory markers. Prognostic scoring analyses showed stepwise increases in MELD, Maddrey’s DF, and ABIC scores from non‑cirrhotic to decompensated stages, with high‑risk categories concentrated almost exclusively in decompensated cirrhosis. These results are consistent with prior studies in acute alcoholic hepatitis and chronic ALD populations, where higher MELD and DF scores independently predict short‑term mortality and guide therapeutic decisions, including corticosteroid eligibility and transplant referral [22].

Clinically, the study confirms that conventional markers, bilirubin, albumin, INR, hemoglobin, platelet count, sodium, and validated scores such as MELD and DF, remain reliable and readily accessible indicators of disease severity in ALD. Within this context, the observed numerical decline and higher prevalence of hypomagnesemia in advanced stages, even without statistical significance, point toward magnesium as a potentially useful, low‑cost adjunct biomarker rather than a standalone severity measure. Given experimental and clinical evidence that magnesium deficiency amplifies oxidative stress, enhances inflammatory responses, and may worsen hepatocellular injury, routine assessment and correction of magnesium in hospitalized ALD patients appears justified. This study suggests that magnesium is biologically relevant in the ALD continuum but also indicates that its clinical utility may lie in complementing, rather than replacing, established prognostic markers, especially in small, single‑center cohorts. Patient follow-up and assessment of progression of ALD stages over time were not performed in this cross-sectional study. Routine assessment and correction of magnesium in hospitalized ALD patients appears justified.

Limitations

A limitation of the study is that it is a single-center study. Thus, the sample size for the compensated cirrhosis group was small, which may have impacted the statistical power of the analysis. Data on determinants of serum magnesium levels, including dietary magnesium intake, duration of alcohol abstinence, medication use, and urinary magnesium loss, were not evaluated. Serum magnesium levels may or may not correlate with intracellular or total body magnesium stores, and, thus, there is the potential to actually underestimate the degree of magnesium deficiency that occurs in advanced liver disease.

## Conclusions

The reliability of conventional severity markers was confirmed in this cross-sectional cohort of 80 patients with ALD, as increasing disease severity was associated with worsening hematological indices, coagulation parameters, liver biochemistry, electrolyte profile, ultrasonographic findings, and prognostic scores. Serum magnesium levels showed a progressive decline with advancing disease severity, although the association was not statistically significant. Hypomagnesemia was more common in severe disease, suggesting a possible role in disease progression and metabolic disturbances. These findings indicate that serum magnesium may serve as a simple and inexpensive adjunct biomarker in ALD. Further large-scale prospective studies are needed to establish its prognostic and therapeutic significance.
